# Longitudinal changes in prospective memory and their clinical correlates at 1-year follow-up in first-episode schizophrenia

**DOI:** 10.1371/journal.pone.0172114

**Published:** 2017-02-28

**Authors:** Fu-Chun Zhou, Chuan-Yue Wang, Gabor S. Ungvari, Chee H. Ng, Yan Zhou, Liang Zhang, Jingjing Zhou, David H. K. Shum, David Man, Deng-Tang Liu, Jun Li, Yu-Tao Xiang

**Affiliations:** 1 Beijing Key Laboratory of Mental Disorders, Beijing Anding Hospital, Capital Medical University, Beijing, China; 2 The University of Notre Dame Australia / Marian Centre, Perth, Australia; 3 School of Psychiatry & Clinical Neurosciences, University of Western Australia, Perth, Australia; 4 Department of Psychiatry, University of Melbourne, Melbourne, Victoria, Australia; 5 Menzies Health Institute Queensland and School of Applied Psychology, Griffith University, Gold Coast, Queensland, Australia; 6 Department of Rehabilitation Sciences, Hong Kong Polytechnic University, Hong Kong SAR, China; 7 Department of Psychiatry, Shanghai Mental Health Center, Shanghai Jiao Tong University School of Medicine, Shanghai, China; 8 State Key Laboratory of Cognitive Neuroscience and Learning, Beijing, China; 9 IDG/McGovern Institute for Brain Research, Beijing, China; 10 Center for Collaboration and Innovation in Brain and Learning Sciences, Beijing Normal University, Beijing, China; 11 Unit of Psychiatry, Faculty of Health Sciences, University of Macau, Macao SAR, China; Nanjing Normal University, CHINA

## Abstract

This study aimed to investigate prospective memory (PM) and the association with clinical factors at 1-year follow-up in first-episode schizophrenia (FES). Thirty-two FES patients recruited from a university-affiliated psychiatric hospital in Beijing and 17 healthy community controls (HCs) were included. Time- and event-based PM (TBPM and EBPM) performances were measured with the Chinese version of the Cambridge Prospective Memory Test (C-CAMPROMPT) at baseline and at one-year follow-up. A number of other neurocognitive tests were also administered. Remission was determined at the endpoint according to the PANSS score ≤ 3 for selected items. Repeated measures analysis of variance revealed a significant interaction between time (baseline vs. endpoint) and group (FES vs. HCs) for EBPM (*F*_(1, 44)_ = 8.8, *p* = 0.005) and for all neurocognitive components. Paired samples *t*-tests showed significant improvement in EBPM in FES (13.1±3.7 vs. 10.3±4.8; *t* = 3.065, *p* = 0.004), compared to HCs (15.7±3.6 vs. 16.5±2.3; *t* = -1.248, *p* = 0.230). A remission rate of 59.4% was found in the FES group. Analysis of covariance revealed that remitters performed significantly better on EBPM (14.9±2.6 vs. 10.4±3.6; *F*_(1, 25)_ = 12.2, *p* = 0.002) than non-remitters at study endpoint. The association between EBPM and 12-month clinical improvement in FES suggests that EBPM may be a potential neurocognitive marker for the effectiveness of standard pharmacotherapy. Furthermore, the findings also imply that PM may not be strictly a trait-related endophenotype as indicated in previous studies.

## Introduction

Cognitive impairment is a core feature of schizophrenia [1–3]. A broad range of deficits in psychomotor speed, memory, attention, reasoning, and social cognition have been reported [4]. Cognitive deficits usually exist even before the onset of illness [3, 5–8], worsen during the early phase of schizophrenia [9], and persist throughout the whole life of patients, which, in turn, influence functional outcomes [10, 11].

The course and trajectory of neurocognitive deficits in schizophrenia remain unclear. Over time, they may deteriorate, show no significant change or even improve in certain cognitive domains [12, 13]. The discrepancy between studies may be due to the heterogeneity of the illness and the types of treatment [14, 15].

A meta-analysis of longitudinal studies has found that schizophrenia patients showed less improvement over time compared to controls in most cognitive variables except on the Stroop Color-Word Test. These results suggest distinctive trajectories of changes in certain cognitive components [13].

Prospective memory (PM) refers to remembering to perform a planned action or intention at some future point in time [16]. PM is thought to play an important role to maintain daily functioning. PM deficits have been consistently confirmed in both chronic [17–26] and first-episode schizophrenia (FES) [27–29]. Non-psychotic first-degree relatives of schizophrenia patients show similar but attenuated PM impairments compared to patients suggesting that PM deficits may be an endophenotype of the illness [30].

There are two subtypes of PM: time-based (TBPM) and event-based PM (EBPM). In EBPM, the carrying out of an intended action is prompted by an external cue, while TBPM relies on the ability to perform an action at a specified time in the future [31]. PM is related to prefrontal-lobe functions in both schizophrenia patients and their first-degree relatives, but TBPM and EBPM may involve different neurocognitive processes [28, 30].

Longitudinal studies on PM changes in FES are important for a better understanding of the pathophysiology of schizophrenia. Certain critical brain areas, such as the frontal pole, hippocampus, lateral prefrontal and inferior parietal regions, involved in PM impairment, have also been reported to be abnormal in schizophrenia [32–38]. To the best of our knowledge, only one follow-up study tracked PM changes over a one-year period finding that while TBPM impairment was unchanged, EBPM significantly improved at the endpoint [39]. Because the control group was not followed up, the impact of a differential practice effect on the results could not be ruled out. It has been suggested that similar practice effects usually occur in both control and clinical groups [40]. In order to control the potential practice effects, both patient and control groups should be followed up [41].

The present study aimed to examine PM changes at one-year follow-up in both FES and healthy controls, and explore their associations with demographic and clinical characteristics.

## Methods

### Participants and study setting

The study was conducted between January 2008 and December 2010 at the National Clinical Research Centre of Mental Disorders located in Beijing Anding Hospital, an 800-bed university-affiliated psychiatric center. Age-, gender- and education level-matched controls were recruited from the community via media advertisement.

In- and out-patients receiving treatment for first-episode psychosis were consecutively referred by their treating psychiatrists to the research team for screening of eligibility. Inclusion criteria were (1) age between 16 and 45 years; (2) Chinese ethnicity; (3) at least six years of education; (4) first episode of the illness; (5) diagnosis of schizophrenia was made according to the DSM-IV (APA, 1994) by two attending psychiatrists who administered the Structural Clinical Interview for DSM-IV (SCID-DSM-IV; First et al., 1996), augmented by a chart review; (6) ability to understand the aims of the study and the contents of the clinical interview and (7) willingness to provide informed consent; (8) either antipsychotic treatment naïve or treatment initiation of less than one month. Patients with a history of drug/alcohol abuse, ECT in the past 12 months, medical or neurological condition(s), or mental retardation were excluded from the study.

The study protocol was approved by the Clinical Research Ethics Committee of Beijing Anding Hospital. Written consent was obtained prior to assessment from patients or their family members for patients younger than 18 years of age as long as they verbally agreed to participate.

### Assessment

Basic socio-demographic and clinical characteristics were collected with a standard form designed for this study by reviewing the charts supplemented by a clinical interview conducted by a psychiatrist. Psychopathology was assessed using the Chinese version of the Positive and Negative Syndrome Scale (PANSS; [42]). In this study, the following five clusters of the PANSS were used: 1. Anergia (N1, N2, G7, G10); 2. Thought disturbance (P2, P3, P5, G9); 3. Activation (P4, G4, G5); 4. Paranoid/belligerence (P6, P7, G8); 5. Depression (G1, G2, G3, G6) [43]. Clinical remission was defined by a PANSS score = /<3 on each of the following items at the endpoint of the study: delusions (P1), unusual thought contents (G9), hallucinatory behavior (P3), conceptual disorganization (P2), mannerism/posturing (G5), blunted affect (N1), social withdrawal (N4) and lack of spontaneity (N6) [44, 45].

The locally validated, Chinese version of the Cambridge PM Test (C-CAMPROMPT [46, 47] was used to assess PM functions for all participants. C-CAMPROMPT is an ecologically-valid PM psychometric test that includes three TBPM and three EBPM tasks while performing a few ongoing activities (i.e., a general knowledge quiz or word-finding puzzle) during a 20 minute period. Participants are allowed to use strategies, such as reminders to assist prospective remembering [48]. The C-CAMPROMPT generates scores on all six tasks, each scoring a maximum of 6, thus the sum score ranges from 0 to 36.

Other neuropsychological assessments also included the following retrospective memory and prefrontal lobe functions:

1. The Hopkins Verbal Learning Test-revised, Chinese version (HVLT-R; [49]) has three learning trials (immediate recall) and a delayed recall subtest, for assessing retrospective memory;
2. The Verbal Fluency Test, Chinese version (VFT; [49]) is composed of two character (phonemic) and two category (sematic) tests; the sum of words produced in the two character and two category trials is averaged and recorded separately;
3. The Color Trails Test (CTT; [49]) comprises two parts (CTT-1 and CTT-2), and is a “culture-fair” version of the Trail Making Test (TMT) for assessing sustained visual attention;
4. The Stroop Color Word Test Chinese version (SCWT; [49]) assesses selective attention and cognitive flexibility; the Stroop Color—Word Interference score was used in this study to measure the ability related to the suppression of a habitual response in favor of an unusual one.

### Procedures

All FES patients were treated with antipsychotic monotherapy during the one-year study period and only short-term (usually less than one week) injectable haloperidol was allowed in agitated patients. For agitation, anxiety and insomnia, short-acting benzodiazepines were used sparingly. In addition, low dose anticholinergic medication (trihexyphenidyl, maximum 6 mg/day) and propranolol were allowed to treat extrapyramidal side effects for any length of time.

All the cognitive evaluation was conducted in a quiet room in the morning at the hospital. In order to minimize the possibility of order effects, neurocognitive functions other than PM were administered in a randomized order followed by the C-CAMPROMPT. All the cognitive tests including C-CAMPROMPT were administered by a psychiatrist (FCZ) and a research nurse who received training in using these instruments. Psychiatric symptoms were evaluated by four other psychiatrists who were blinded to the patients’ performance on the cognitive tasks. The inter-rater reliability exercise of the PANSS subscales yielded satisfactory agreement; the intra-class correlation coefficients (ICC) ranged from 0.83 to 0.86.

### Data analysis

All analyses were performed using SPSS Version 20.0. Comparisons between patients and controls, and between clinically remitted patients and those who failed to remit (‘remitters’ and ‘non-remitters’, respectively) with regard to clinical variables were conducted by independent sample *t*-test and Chi square test, as appropriate. Paired samples t-tests were used to compare the neurocognitive variables including TBPM and EBPM between entry and endpoint in both patients and controls.

Repeated measures analysis of variance (ANOVA) was performed for each cognitive test with group (patient vs. control; remitters vs. non-remitters) as the between-group factor, and time (baseline vs. 1-year) as the within-group factor. Effects of time, group, and the interaction between time and group were examined. Cognitive domains that exhibited significant time*group effects in patients and HCs were also examined using paired samples *t*-tests to investigate longitudinal changes. In addition, analysis of covariance (ANCOVA) was performed in the patient group comparing cognitive performance between remitters and non-remitters at endpoint with age, gender, educational level and baseline PM score as covariates. All statistical tests were two-tailed. Level of significance was set at the 0.05.

## Results

Of the 55 FES patients screened for eligibility, 47 fulfilled entry criteria and participated in the study, but only 40 completed all the assessments at baseline. Eight patients did not complete the endpoint assessment due to lack of interest to continue participation. Patients who dropped out did not differ significantly from those who completed the follow-up in terms of age, gender and education level. Only the 32 patients who completed the endpoint assessment at 1-year follow-up were included for analyses.

At baseline, 10 patients were drug-naive for antipsychotics, while the other 22 patients had received less than 1 month of antipsychotic monotherapy with either risperidone, olanzapine, aripiprazole, quetiapine, or haloperidol. No patients had received anticholinergic medications before the study. Six patients received low dose (0.5mg-1mg daily) lorazepam before the study entry.

### Comparison between patients and controls regarding longitudinal changes in PM and other cognitive functions

Table 1 shows the basic demographic and clinical characteristics of the study sample by groups at baseline and endpoint. At baseline, patients performed significantly poorer than controls in TBPM (*F*_(1,44)_ = 6.5, *p* = 0.014), EBPM (*F*_(1,44)_ = 31.6 *p*<0.001), HVLT-R (*F*_(1,44)_ = 6.2, *p* = 0.017), CTT-1 (*F*_(1,44)_ = 8.7, *p* = 0.005), SCWT (*F*_(1,44)_ = 7.9, *p* = 0.007) and VFL (*F*_(1,44)_ = 5.7, *p* = 0.021) after controlling for age, gender and educational level. At endpoint, patients’ score remained significantly lower than controls in TBPM (*F*_(1,44)_ = 5.1, *p* = 0.029), EBPM (*F*_(1,44)_ = 6.3, *p* = 0.015), HVLT-R (*F*_(1,44)_ = 7.0, *p* = 0.011), CTT-1(*F*_(1, 44)_ = 18.9, *p*<0.001), CTT-2 (*F*_(1,44)_ = 12.4, *p* = 0.001), SCWT (*F*_(1,44)_ = 8.8, *p* = 0.005), VFL (*F*_(1,44)_ = 21.4, *p*<0.001) and VFC (*F*_(1,44)_ = 13.7, *p* = 0.001) after controlling for age, gender and education level.

**Table 1:** Comparions of longitudinal changes between FES patients and controls with respect to PM and other cognitive functions.

	FES (n = 32)							Controls (n = 17)							#Comparison between groups		
	At baseline		At endpoint		Statistics			At baseline		At endpoint		Statistics			Statistics		
	N	%	N	%	χ^2^	df	p	N	%	N	%	χ^2^	df	p	χ^2^	df	p
Male sex	19	59.4	—	—	—	—	—	13	76.5	—	—	—	—	—	1.4	1	0.23
	Mean	SD	Mean	SD	T/Z	df	P	Mean	SD	Mean	SD	T/Z	df	P	F/Z	df	P
Age (years)	26.2	8.1	—	—	—	—	—	25.5	5.6	—	—	—	—	—	-0.5	—^a^	0.61
Education (years)	13.5	2.2	—	—	—	—	—	12.6	2.3	—	—	—	—	—	-1.3	—^a^	0.20
HVLT-R	22.5	6.5	24.4	4.9	-2.5	31	0.02	26.8	5.3	27.3	4.4	-0.5	16	0.60	0.7	1, 44	0.40
CTT-1	56.9	17.4	51.6	14.1	1.8	31	0.09	43.3	15.1	35.4	9.0	1.8	16	0.09	1.2	1, 44	0.67
CTT-2	98.7	54.4	97.3	41.7	-0.2	—^a^	0.87	76.0	20.1	70.7	13.4	-0.9	—^a^	0.37	0.7	1, 44	0.41
SWCT	34.1	9.8	33.6	8.4	0.3	31	0.79	40.1	7.1	41.9	12.8	-0.6	—^a^	0.57	0.3	1, 44	0.58
VFL	2.6	1.0	1.8	1.5	-2.7	—^a^	0.007	3.6	1.7	4.1	2.0	-1.2	—^a^	0.24	8.0	1, 44	0.007
VFC	12.8	3.7	12.5	2.8	0.5	31	0.63	14.0	4.4	14.5	2.3	-0.5	16	0.62	1.5	1, 44	0.23
TBPM	7.9	5.2	9.3	5.3	-2.2	31	0.03	11.5	5.4	12.6	4.7	-0.7	—^a^	0.48	0.1	1, 44	0.74
EBPM	10.3	4.8	13.1	3.7	-3.1	31	0.004	16.5	2.3	15.7	3.6	-1.1	—^a^	0.26	8.8	1, 44	0.005

Note: (1) Results reported in this column (#) include the comparisons between the two groups regarding baseline age, gender, educational level and longitudinal changes in PM and other cognitive functions. (2) For the repeated measures analysis of variance, only the effect of time*group interaction was presented in the table.

^a^ = Wilcoxon signed rank test; TBPM = time-based prospective memory; EBPM = event-based prospective memory; HVLT-R = Hopkins Verbal Learning Test-Revised Version; VFL = Verbal Fluency Test (letter test); VFC = Verbal Fluency Test (category test); CTT = Color Trails Test; SCWT = Stroop Color Word Test;

In the patient group paired samples *t*-tests revealed significant changes from baseline to the endpoint on TBPM, EBPM, HVLT-R and VFL scores. No difference was found between baseline and endpoint assessments in the control group in any of the cognitive performances including TBPM and EBPM.

After controlling for age, gender and education level, results of a repeated measures ANOVA revealed significant time (baseline vs. endpoint)*group (FES vs. HCs) interaction only in EBPM (*F*_(1,44)_ = 8.8, *p* = 0.005) (Table 1).

### The association between clinical outcomes and neurocognitive trajectories

**Fig 1:**
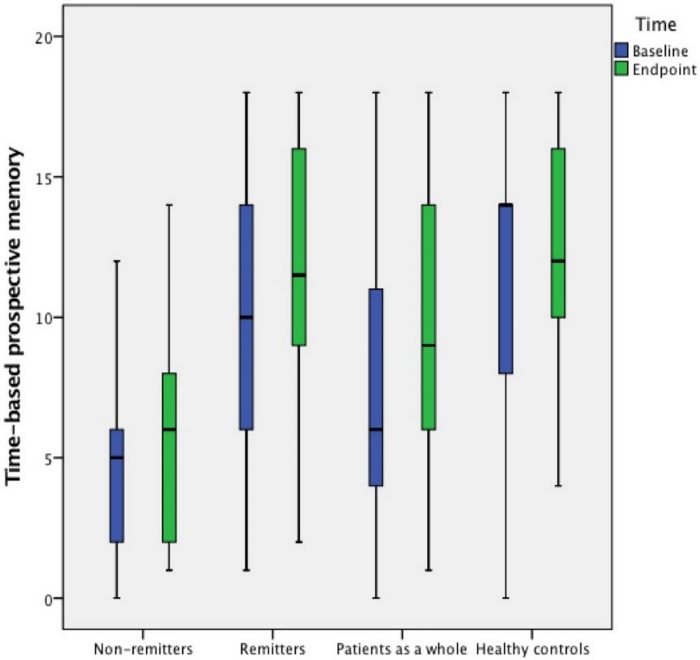
Time-based PM in FES patients and healthy controls.

**Fig 2:**
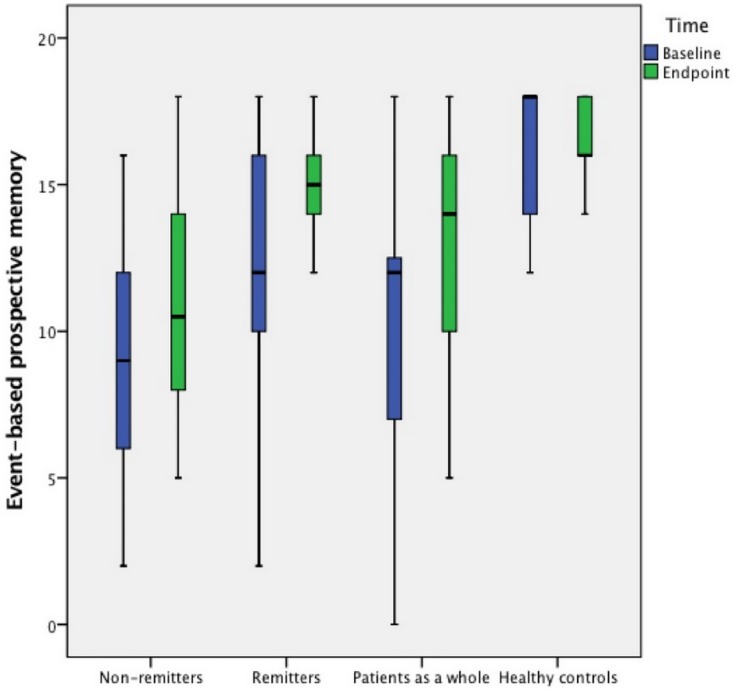
Event-based PM in FES patients and healthy controls.

Patients’ demographic and clinical characteristics and **neurocognitive performance** by remission status are shown in Table 2 and Figs 1 and 2. At the endpoint, there were 19 (59.4%) remitters. Compared to non-remitters, remitters had a significantly shorter duration of illness, a better performance on HVLT-R and TBPM, and lower PANSS “Anergia” score at baseline; while having better performance on TBPM and EBPM, lower PANSS “Anergia” and “Thought disturbance” scores at endpoint.

**Table 2:** Comparison between remitters and non-remitters with respect to demographic, clinical, PM and other cognitive variables at baseline and endpoint.

	At baseline							At endpoint						
	Remitters (n = 19)		Non-remitters (n = 13)		Statistics			Remitters (n = 19)		Non-remitters (n = 13)		Statistics		
	N	%	N	%	χ^2^	df	p	N	%	N	%	χ^2^	df	p
Male sex	11	57.9	8	61.5	—^b^	—	1.00	—	—	—	—	—	—	—
Inpatients	6	31.6	4	30.8	—^b^	—	1.00	0	0.0	0	0.0	—	—	—
Medication status														
Drug naïve	6	31.6	4	30.8	—^b^	—	1.00	0	0.0	0	0.0	—	—	0.00
risperidone	3	15.8	3	23.1	—^b^	—	0.67	5	26.3	3	23.1	—^b^	—	1.00
olanzapine	0	0.0	1	7.7	—^b^	—	0.40	5	26.3	5	38.5	—^b^	—	0.70
aripiprazole	7	36.8	3	23.1	—^b^	—	0.47	9	47.4	5	38.5	—^b^	—	0.73
quetiapine	2	10.5	0	0.0	—^b^	—	0.50	0	0.0	0	0.0	—	—	0.00
haloperidol	1	5.3	2	15.4	—^b^	—	0.55	0	0.0	0	0.0	—	—	0.00
Concomitant medications														
Anticholinergics	0	0.0	0	0.0	—	—	—	6	31.6	6	46.2	—^b^	—	0.47
Benzodiazepines	3	15.8	3	23.1	—^b^	—	0.67	0	0.0	0	0.0	—	—	—
	Mean	SD	Mean	SD	T/Z	df	P	Mean	SD	Mean	SD	T/Z	df	P
Age (years)	25.2	8.5	27.6	7.5	-1.2	—^a^	0.23	—	—	—	—	—	—	—
Education (years)	13.1	2.4	14.0	1.7	-1.0	—^a^	0.32	—	—	—	—	—	—	—
Duration of illness (months)	8.1	4.6	20.0	11.1	-3.1	—^a^	0.002	—	—	—	—	—	—	—
PANSS subscales														
Positive	27.3	6.9	29.8	6.4	-1.0	30	0.31	7.6	1.3	10.9	4.0	-3.2	—a	0.002
Negative	28.1	4.3	25.5	4.1	1.7	30	0.11	9.2	2.9	16.5	5.3	-3.8	—a	<0.001
General	41.3	5.63	42.5	6.57	-0.6	30	0.58	18.4	2.3	23.2	4.8	-4.4	16.8	<0.001
PANSS cluster														
Anergia	9.8	3.0	12.1	3.1	-2.1	30	0.04	5.1	1.4	7.8	2.6	-3.4	16.4	0.003
Thought disturbance	13.8	2.8	14.9	2.7	-1.2	30	0.26	4.4	0.8	6.3	2.4	-2.7	—^a^	0.02
Activation	7.2	1.7	6.2	2.4	1.4	30	0.19	3.1	0.5	3.9	1.5	-1.9	—^a^	0.22
Paranoid/belligerence	10.0	1.7	8.8	2.5	1.7	30	0.11	3.1	0.2	4.0	1.5	-2.4	—^a^	0.10
Depression	8.5	2.9	8.7	3.6	-0.2	30	0.85	4.6	1.5	4.8	2.0	-0.1		0.94
CPZeq (mg)	67.6	63.4	84.7	84.3	-0.5	—^a^	0.63	267.6	68.6	292.3	81.3	-0.7	—^a^	0.47
HVLT-R	24.6	5.8	19.3	6.3	2.5	30	0.02	25.5	4.9	22.9	4.7	1.6	30	0.13
CTT-1	58.1	19.8	55.2	13.7	0.5	30	0.64	50.6	14.1	53.0	14.6	-0.5	30	0.64
CTT-2	102.6	69.6	93.0	18.2	-0.3	—^a^	0.79	95.8	42.1	99.4	42.7	-0.6	—^a^	0.56
SWCT	35.3	10.5	32.5	8.9	0.8	30	0.44	35.7	6.3	30.5	10.3	1.8	30	0.09
VFL	2.4	0.9	3.0	1.1	-1.6	—^a^	0.12	2.0	1.5	1.4	1.4	-1.0	—^a^	0.33
VFC	13.7	4.0	11.6	3.0	1.6	30	0.12	12.9	2.7	11.9	2.9	1.0	30	0.32
TBPM	10.0	4.9	4.9	4.2	3.1	30	0.005	11.7	4.6	5.7	4.1	3.8	30	<0.001
EBPM	11.3	5.0	8.9	4.2	1.4	30	0.17	14.9	2.6	10.4	3.6	-3.3	—^a^	<0.001

^a^ = Mann-Whitney U test;

^b^ = Fisher's Exact Test; PANSS = Positive and Negative Syndrome Scale; TBPM = time-based prospective memory; EBPM = event-based prospective memory; HVLT-R = Hopkins Verbal Learning Test-Revised Version; VFL = Verbal Fluency Test (letter test); VFC = Verbal Fluency Test (category test); CTT = Color Trails Test; SCWT = Stroop Color Word Test; CPZeq = chlorpromazine equivalent

After controlling for age, gender, educational level, duration of illness and baseline EBPM score, remitters still performed significantly better on EBPM (*F*_(1, 25)_ = 12.2, *p* = 0.002) than non-remitters at endpoint. However, the difference on TBPM (*F*_(1, 25)_ = 3.1, *p* = 0.09) scores between remitters and non-remitters disappeared after basic demographic characteristics and the baseline assessments of cognitive functions were controlled for.

## Discussion

To the best of our knowledge, this is the first longitudinal, healthy controlled study on PM in first-episode schizophrenia. The main finding is that EBPM significantly improved over time in FES. Patients who remitted from their first-episode performed significantly better on EBPM than non-remitters at 1-year follow-up suggesting that the improvement in EBPM performance is probably associated with clinical remission in FES. These findings are consistent with those of another longitudinal study that explored changes in PM in FES [39]. In that study, patients’ EBPM performance gradually improved over time and eventually showed no significant difference compared to healthy controls at the 1-year assessment. However, the control group was not followed up and tested at 1-year, therefore the practice effect on the results could not be excluded since TBPM deficit remained at the endpoint. In addition, PM was assessed using a dual-task paradigm in the Cheung **e**t al.’s study [39]; i.e., participants needed to execute the PM task at a certain time or on the appearance of certain PM cues. In the present study, ecologically valid paradigms were used. The Cambridge PM Task has the advantage of simulating real-life situations and allowing participants to adopt strategies, such as taking notes as reminders, to facilitate PM performance. Moreover, only the PANSS was used to measure psychopathology in the Cheung et al.’s study; in contrast, both the PANSS and remission using the Andreasen’s definition [44, 45] were used in this study. Finally, types and doses of antipsychotics were included in this study.

Neuroimaging studies found that the prefrontal cortex, particularly the lateral and medial parts, plays an important role in generating PM [50–54]. In addition, the left parahippocampal gyrus and the middle temporal gyrus are also activated during PM tasks in PET and fMRI studies [50, 55–57]. Furthermore, both the prefrontal and temporal cortices have been impaired in schizophrenia patients and their first-degree relatives [58, 59]. Taken together, these findings suggest that impairments of PM might be an endophenotype of schizophrenia. Endophenotypes are considered as “quantifiable biological variations or deficits that are types of stable trait markers or indicators of presumed inherited vulnerability or liability to a disease” [60]. However, the relative stability does not mean that the endophenotype could not be changed in response to treatment. In clinical practice the primary focus is not to modify the genetic variations associated with the endophenotype, but to “normalize” neural circuits and activate collateral circuits [61], which may play a role in restoring neurocognitive and neurophysiological functions.

Findings on the trajectory of neurocognitive functioning in schizophrenia are inconsistent. For example, verbal memory and learning were reported to be worsened, unchanged, or improved across different studies [62–67]. In one study, there was a significant decline in Verbal Learning and improvement on Reasoning/Problem Solving and Social Cognition at 2-year follow-up in FES patients, but not in the control group, indicating a different neurocognitive trajectory [68].

The inconsistency of the findings may reflect the involvement of different neural circuits in cognitive processes. There have been several neuroimaging and neuropsychological studies on the neural circuits possibly involved in TBPM and EBPM. A positron emission tomography (PET) study has found the activation differences in rostral prefrontal cortex between TBPM and EBPM tasks [56]. Specifically, when carrying out TBPM tasks, participants showed more activation in areas of left superior frontal gyrus, right superior frontal gyrus, anterior medial frontal lobe and anterior cingulate gyrus. When carrying out EBPM tasks, a different region in the left superior frontal gyrus was found to be more active. Using the human lesion approach, Volle et al. (2011) found that TBPM deficit in both words and pictures was specifically associated with lesions in the right polar prefrontal region, which was not due to impairments in basic neurocognitive functions. TBPM and EBPM may involve different frontal lobe processing in FES; specifically, TBPM was found to be independently associated with CTT-2 and WCST-CC, whereas EBPM was found to be predicted by WCST-PE [28].

It is thought that TBPM requires self-initiated retrieval and places greater demand on the prefrontal cortex than EBPM [69]. This assumption was confirmed through moderator analysis in a meta-analysis [70], showing that the variances were heterogeneous between TBPM and EBPM; TBPM being more impaired than EBPM in schizophrenia. In a longitudinal study only TBPM predicted remission after 8-week treatment pointing to the possible role of TBPM in the short-term outcome of FES [71].

EBPM exhibited greater improvement in patients than in controls, while TBPM remained impaired in patients, which is consistent with earlier findings [39]. In addition, the present study found that remission is positively associated with better EBPM performance. These results may have the following theoretical and clinical implications: (1) TBPM impairment is relatively stable suggesting that it is more likely to be an endophenotype and associated to genetic disposition; (2) EBPM could be potentially a neurocognitive marker of treatment response in first episode schizophrenia.

The strengths of this study include the use of standardized assessment of PM, widely accepted criteria of remission and standardized antipsychotic monotherapy. However, the results should be interpreted with caution due to the following methodological limitations. First, only a third of patients were drug-naive at baseline and some patients received anticholinergics or benzodiazepines. Second, the 1-year study period is relatively short; therefore, longer term emerging changes in cognitive components could not be detected. Third, the sample size was relatively small, which limited the statistical power. Fourth, ideally a control group of psychotropic drug-naive first-episode schizophrenia patients should be included in the one-year follow-up. However, this would have been clinically impossible and unethical. Fifth, due to logistic reasons, neuroimaging and electrophysiological measures were not included in this study. Finally, the types and doses of antipsychotic medications used in the study period were not fixed, which might have influenced the clinical outcomes. In order to examine the underlying neural circuits associated with PM changes in schizophrenia, longitudinal studies with fixed-dose antipsychotic monotherapy should be conducted coupled with extended follow-up and neuroimaging and electrophysiological measures.

In conclusion, at 1-year follow-up, only EBPM improvement was associated with clinical remission suggesting that it may be a potential neurocognitive marker for treatment response. PM is likely to be a heterogeneous cognitive function both from theoretical and clinical viewpoints in that TBPM appears to be a trait-related endophenotype, while EBPM may possibly be a state-related component of PM.
